# Role of nitrite in the competition between denitrification and DNRA in a chemostat enrichment culture

**DOI:** 10.1186/s13568-017-0398-x

**Published:** 2017-05-11

**Authors:** Eveline M. van den Berg, Julius L. Rombouts, J. Gijs Kuenen, Robbert Kleerebezem, Mark C. M. van Loosdrecht

**Affiliations:** Department of Biotechnology, Delft University of Technology, Van der Maasweg 9, 2629 HZ Delft, The Netherlands

**Keywords:** Chemostat, Denitrification, Dissimilatory nitrate reduction, DNRA, Enrichment

## Abstract

Denitrification and dissimilatory nitrate reduction to ammonium (DNRA) are two microbial processes that compete for oxidized nitrogen compounds in the environment. The objective of this work was to determine the role of nitrite versus nitrate as terminal electron acceptor on the competition between DNRA and denitrification. Initially, a mixed culture chemostat was operated under nitrate limitation and performed DNRA. Stepwise, the influent nitrate was replaced with nitrite until nitrite was the sole electron acceptor and N-source present. Despite changing the electron acceptor from nitrate to nitrite, the dominant process remained DNRA and the same dominant organism closely related to *Geobacter lovleyi* was identified. Contrary to previous studies conducted with a complex substrate in marine microbial communities, the conclusion of this work is that nitrate versus nitrite as electron acceptor does not generally control the competition between DNRA and denitrification. Our results show that the effect of this ratio must be interpreted in combination with other environmental factors, such as the type and complexity of the electron donor, pH, or sulfide concentrations.

**Electronic supplementary material:**

The online version of this article (doi:10.1186/s13568-017-0398-x) contains supplementary material, which is available to authorized users.

## Introduction

Nitrate reduction is an important process in the nitrogen cycle. Nitrate can be reduced to nitrogen gas by denitrification, which removes the nitrogen from the ecosystem. This process balances natural and anthropogenic nitrogen inputs and counteracts eutrophication. Alternatively, dissimilatory nitrate reduction to ammonium (DNRA) retains the nitrogen in the environment. For instance, in agricultural soils, this retention can lead to a more optimal use of nitrogen containing fertilizer and prevention of nitrate leaching (Silver et al. 2001). Finally, the autotrophic anaerobic ammonium oxidation (anammox) bacteria can also reduce nitrate to nitrite and ammonium and subsequently to dinitrogen gas, but is not considered relevant in carbon source abundant enrichments. As the fate of nitrate can have important implications for the ecosystem (Burgin and Hamilton 2007; Kraft et al. 2011), as well as the successful operation of wastewater treatment systems, we want to understand the competition between DNRA and heterotrophic denitrification in order to allow manipulation of nitrate reduction towards the desired end product (N_2_ or NH_4_^+^).

Denitrification was long assumed to be the dominant nitrate reduction process in the environment. DNRA had received relatively little attention, in particular with respect to its quantitative contribution to the nitrogen cycle. In the past decade, DNRA has become recognized to contribute significantly to nitrate reduction in the environment (Brin et al. 2015; Decleyre et al. 2015; Giblin et al. 2013; Rütting et al. 2008). We have limited understanding of the environmental factors that control the nitrate reduction processes (Jetten 2008; Kraft et al. 2011). A known important factor in the competition for nitrate between DNRA and denitrification is the ratio of available electron donor (i.e. easily degradable carbon) and electron acceptor (i.e. nitrate or nitrite) (Kraft et al. 2014; van den Berg et al. 2016; Yoon et al. 2015a). Consistently, in organic carbon rich environments, where nitrate is limiting, DNRA dominates, and in environments with excess of nitrate and limiting carbon denitrification dominates (Rütting et al. 2011; Tiedje et al. 1982).

Kraft et al. (2014) postulated that the terminal electron acceptor has a determinative effect on the competition. In DNRA and denitrification, nitrate and nitrite can both be terminal electron acceptors. Kraft and her colleagues enriched nitrite reducers from marine sediments on a complex carbon source, including amino acids, sugars and organic acids, in a continuous fed chemostat enrichment system and observed only conversion to nitrogen gas and no production of ammonia. When nitrate was the electron acceptor instead of nitrite, the mixed enrichment cultures showed combined fermentative and respiratory properties with a predominant conversion of nitrate to ammonia. These observations were attributed to a comparatively higher apparent affinity of denitrifiers for nitrite and a comparatively higher apparent affinity of DNRA bacteria for nitrate. Kraft et al. (2014) concluded therefore that supply of nitrate/nitrite was a key controlling factor in the nitrate partition. Interestingly and alternatively, Yoon et al. (2015b) reported an opposite trend in *Shewanella loihica* chemostat cultures with nitrate or nitrite as electron acceptor. Nevertheless, they also conclude that nitrite is a determining factor in the choice between DNRA or denitrification.

As the effect of nitrite as a controlling factor in the competition between the two nitrate reducing processes is ambiguous, we wanted to verify the determinative effect of nitrate versus nitrite using an enrichment culture grown on acetate mineral medium in continuous culture. With our simplified system we can obtain additional and more quantitative insight in the DNRA process. Since the acetate in our culture is directly oxidized to carbon dioxide, the system is better defined than the more complex, partly fermentative marine microbial community studied by Kraft et al. (2014), which even showed significant turnover of sulfate and sulfide. Since we used enrichment cultures, our study is an important complement to the pure culture studies by Yoon et al. (2015b) with *S. loihica*, which was not isolated based on DNRA capacity.

In this work we describe the results obtained with a chemostat culture inoculated with activated sludge and operated with freshwater-mineral medium containing acetate as electron donor and, initially, nitrate as electron acceptor as described by van den Berg et al. (2015), operated at a dilution rate adequate for growth of both the denitrifying and DNRA bacteria. Throughout the study, the culture was operated under electron acceptor limiting conditions. The initial nitrate-only culture performed DNRA. Stepwise, the influent nitrate was replaced with nitrite until nitrite was the sole electron acceptor and N-source present. Steady state populations were analyzed with Fluorescent In Situ Hybridization (FISH) probes.

## Materials and methods

### Chemostat operation

The experiments were conducted using an open continuous-flow stirred-tank reactor (CSTR, i.e. a flow controlled chemostat). The basic reactor set up was the same as described by van den Berg et al. (2015). The redox potential was monitored using a Redox probe (Mettler Toledo, Tiel, The Netherlands). Before the start of the experiments of this study the reactor had been running continuously for 2 years under the conditions described in this paper. Two separate media flows were supplied to the reactor in equal amounts. Both culture media were autoclaved before use and sparged with a small flow of nitrogen gas while connected to the chemostat to ensure anaerobic conditions. Medium A contained per liter: 22.0 mmol KH_2_PO_4_, 1.2 mmol MgSO_4_·7H_2_O, 1.5 mmol NaOH, 1.5 mg yeast extract (as vitamin supplement) and 5 ml trace element solution (Vishniac and Santer 1957), with only 2.2 g ZnSO_4_·7H_2_O per liter, and varying amounts of NaNO_3_ and/or NaNO_2_ (Table 1). Medium B contained varying concentrations of acetate, NaCH_3_COO·3H_2_O (Table 1), to match the amount of electron acceptor provided. Note that acetate was always in excess, only the residual concentrations were decreased. Each time when the feed to the reactor was changed, 10 ml of activated sludge and 10 ml of an enriched denitrifier community on acetate were added to the reactor culture, to increase the potential for enriching the most competitive organism in the culture. Both media were pumped at 26 ml/h into the reactor so that the total influent was 52 ml/h. The effluent pump was controlled using a level sensor. The resulting dilution rate was 0.027 h^−1^. The culture was assumed to be in steady state if conversions were constant for five doubling times, which was approximately 8 days.

**Table 1:** Nitrate, nitrite and acetate concentrations in the influent as used in the different experimental periods

Days	Concentration in the influent (mM)			N % as nitrite
	Nitrate	Nitrite	Acetate	
0–32	11.8	0.00	22.1	0
33–60	8.83	2.62	22.1	23
61–82	5.88	5.23	20.2	47
83–123	2.94	7.85	16.5	73
124–165	0.00	11.8	14.7	100

### Analytical procedures

Oxygen, carbon dioxide, nitric oxide and nitrous oxide concentrations in the headspace of the reactor were monitored in dried gas using a gas analyzer (NGA 2000, Rosemount, Chanhassen, MN, USA). The flow of nitrogen gas to the reactor was kept at 100 ml/min using a mass flow controller (Brooks Instrument, Ede, The Netherlands), to maintain sufficient flow through the gas analyzer (80 ml/min).

Samples taken from the reactor were centrifuged and supernatants were used for analysis of acetate and nitrogen compounds. The acetate concentration in the liquid phase was measured by High Performance Liquid Chromatography using an Aminex HPX-87H column (*T* = 60 °C) from Bio-Rad Laboratories (Hercules, CA, USA) coupled to a UV and RI detector using phosphoric acid (0.01 M) as eluent. An indication of the nitrite- and nitrate-concentration in the reactor was obtained with test strips (Merck Millipore, Carrigtwohill, Ireland). When this was not zero, the concentrations were measured more accurately. Nitrate-, nitrite- and ammonium-concentrations were determined spectrophotometrically with commercial cuvette test kits (Hach Lange, Düsseldorf, Germany).

To determine the biomass concentration, the reactor effluent was centrifuged (10,000 rpm for 20 min) and the pellet was dried at 105 °C. Subsequently the ash content was subtracted to obtain VSS concentration. The ash content was determined by burning the organic parts of the dried pellet at 550 °C. Protein content of the biomass was measured using the Uptima BC Assay Protein Quantitation Kit (Interchim, Montluçon, France).

The biomass composition was calculated from the measured Total Organic Carbon (TOC) and Total Organic Nitrogen (TON) of washed biomass pellets, using a TOC-L CPH/CPN analyzer (Shimadzu Benelux,’s-Hertogenbosch, The Netherlands). TOC was determined as Total Carbon (TC) subtracted by Inorganic Carbon (IC) (TOC = TC − IC). Biomass composition was measured for several steady states and did not significantly differ for the different populations. In our calculations we used the average of 0.23 ± 0.01 mol N per C-mol biomass.

A balance of degree of reduction and a charge balance of incoming and exiting elements in the chemostat were set up to verify the consistency of our measurements. The concentration of volatile suspended solids (VSS) was used as biomass concentration. The growth in the system is relatively fast compared to, e.g. soils. As a result biomass decay is not significant and immobilization/re-mineralization negligible. Hence, ammonium production was attributed to nitrate reduction by DNRA. As emissions of nitric and nitrous oxide were not detected, the nitrogen not accounted for in ammonium, nitrate, nitrite or biomass was assumed to be converted to N_2_. Sulfide was not detectable (<2 µmol/l). The dissolved CO_2_ species, mostly HCO_3_^−^, which leave the reactor in the effluent, were estimated and taken into account. It was assumed Henry’s law applies, using T = 298 K, p = 1 atm, ${\text{H}}^{\text{cp}}_{{{\text{CO}}_{2} }}$ = 3.4.10–4 mol/(m^3^ Pa) (2), pK_a_ = 6.35 $\left( {{\text{H}}_{2} {\text{CO}}_{3} { \leftrightarrows }{\text{HCO}}_{3}^{ - } + {\text{H}}^{ + } } \right),$ ${\text{K}}_{{{\text{HCO}}_{2} }}$ = 1.7 × 10^−3^ (Housecroft and Sharpe 2005).

### Microbial population analysis

The microbial composition of the culture was analyzed with FISH as described by Johnson et al. (2009), using a hybridization buffer containing 35% (v/v) formamide. The applied probes are listed in Table 2. The general probe mixture EUB338 labeled with Cy5 was used to identify all eubacteria species in the sample. In the shown result, we used the EUB338 (Cy5), the Beta42a probe, labeled with FLUOS (plus an unlabeled Gamma42a probe, to minimize erroneous hybridizations of Beta42a) and GeoBac464, a probe labeled with Cy3 specifically designed for the detection of the 16S rRNA of the DNRA microorganism dominating the culture under nitrate limitation (Additional file 1: Table S1; van den Berg et al. 2015).

**Table 2:** Probes used in FISH analysis of the culture

Probe	Sequence (5′→3′)	Dye	Specificity	Reference
EUB338mix	GCWGCCWCCCGTAGGWGT	Cy5	Most bacteria	Amann et al. (1990) and Daims et al. (1999)
Beta42a	GCCTTCCCACTTCGTTT	Fluos	*Betaproteobacteria*	Manz et al. (1992)
Gamma42a	GCCTTCCCACATCGTTT	None	*Gammaproteobacteria*	Manz et al. (1992)
GeoBac464	AGCCTCTCTACACTTCGTC	Cy3	Specific for DNRA bacterium	van den Berg et al. (2015)

Probes were synthesized and 5′-labeled with either the FLUOS or with one of the sulfoindocyanine dyes Cy3 and Cy5 (Thermo Hybaid Interactiva, Ulm, Germany). Slides were observed with an epifluorescence microscope (Axioplan 2, Zeiss, Sliedrecht, The Netherlands), and images were acquired with a Zeiss MRM camera and compiled with the Zeiss microscopy image acquisition software (AxioVision version 4.7, Zeiss) and exported as TIFF format.

In addition, denaturing gradient gel electrophoresis (DGGE) was performed. Biomass samples were collected from the reactor and centrifuged and stored at −20 °C. The genomic DNA was extracted and analyzed as described by van den Berg et al. (2015). The set of primers used is the 341F (containing a 40-bp GC clamp) and 907R (Schäfer and Muyzer 2001). The obtained sequences were corrected using the program Chromas Lite 2.1.1 (http://technelysium.com.au) and then compared to sequences stored in GenBank using the Basic Local Alignment Search Tool (BLAST) algorithm (http://www.ncbi.nlm.nih.gov/blast).

## Results

**Fig. 1:**
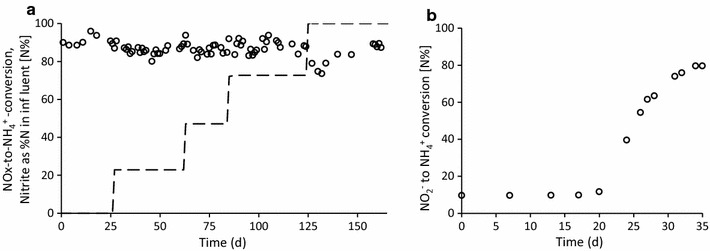
Ammonium production in the acetate fed chemostat systems, as a percentage of the NO_x_ conversion in time. This includes both dissimilatory and assimilatory production of ammonium. **a** Ammonium production (*open circles*) for the varying percentages of NO_2_^−^ in the influent nitrogen (*dashed line*). The other influent nitrogen was nitrate. **b** Ammonium formation in the enrichment inoculated with activated sludge with nitrite as electron acceptor

A chemostat based enrichment system was operated under electron acceptor (nitrate/nitrite) limiting conditions with acetate as electron donor and a dilution rate of 0.027 h^−1^. Acetate was always detected in the effluent of the reactor and the redox potential was constant during the experimentation at minus 480 ± 50 mV. This confirmed electron acceptor limiting conditions. Initially, the electron acceptor was nitrate and the culture converted 70 ± 3% of the influent nitrate-N to ammonium, 15 ± 2% was incorporated into biomass (Additional file 1: Table S2), and 15% was presumably converted to dinitrogen gas. Stepwise, the influent nitrate was replaced by nitrite. In the first step 23% of the nitrate was replaced, and subsequently 47 and 73%, until all influent nitrogen was nitrite. When nitrate in the feed was changed to nitrite, the fraction of the influent N converted to ammonia did not change (Fig. 1a). Thus, despite the change of electron acceptor from nitrate to nitrite, DNRA remained equally dominant in the reactor.

To confirm that DNRA bacteria can not only remain, but also outcompete the denitrifiers with nitrite as electron acceptor, a second reactor was started up in the same conditions as the nitrite-only system. Starting from an inoculum of activated sludge, a DNRA culture was enriched with nitrite as electron acceptor (Fig. 1b). Thus, we confirmed that DNRA bacteria successfully outcompete denitrifiers when nitrite is the limiting electron acceptor in the chemostat enrichment culture.

Both in the nitrate-only and nitrite-only culture, the C/N ratio of the biomass was measured to be the same, 0.23 ± 0.01 molN/molC. The biomass yield was 12.3 ± 1.4 gVSS/mol NO_2_^−^ for growth on nitrite, which was lower than for nitrate, 19.0 ± 0.3 gVSS/mol NO_3_^−^ (Additional file 1: Table S3). The protein content of the VSS was measured to be 0.60 ± 0.04 mg protein/mg VSS. When both yields are compared as per mole electron donor (acetate), the yields are similar for growth on nitrate and nitrite (13.4 ± 0.6 and 11.7 ± 1.5 gVSS/mol acetate respectively).

**Fig. 2:**
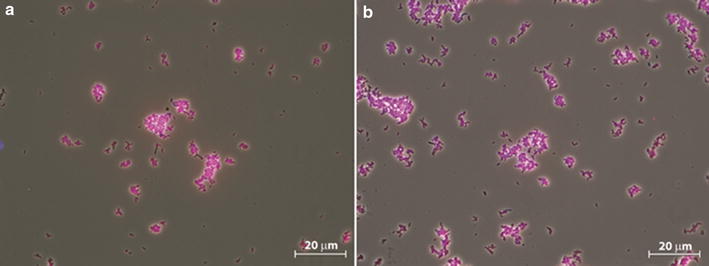
FISH microscopic photographs of steady state cultures. **a** The culture grown on nitrate only. **b** The culture grown on nitrite only. The cells were stained with Cy5-labeled probes for bacteria (EUB338mix, blue), FLUOS-labeled probes for *Betaproteobacteria* (Beta42a, green) and Cy3-labeled probes specific for the reactor species (GeoBac464, red). Cells that are green indicate cells to which the probes EUB338mix and Beta42a were hybridized. Cells that are purple indicate cells to which the probes EUB338mix and GeoBac464 were hybridized

The microbial population analysis using FISH showed that also the population did not change when changing from nitrate to nitrite as electron acceptor in the system (Fig. 2). In Fig. 2 almost all fixed bacteria of both cultures are purple colored and thus almost all bacteria hybridized with a probe specific for one *Geobacter* ribotype (Additional file 1: Table S1), described by van den Berg et al. (2015). Additional DGGE analysis showed the ribotypes were identical (Additional file 1: Figure S1). The green colored bacteria (*Betaproteobacteria*) are not necessarily the same species in both cultures, but are present in the same low amount in both steady states and are therefore assumed not to be relevant for the major conversion stoichiometry.

## Discussion

In our chemostat enrichment system provided with acetate as the simple non-fermentable carbon and energy source, the competition between DNRA and denitrification was unaffected by the type of electron acceptor. Despite changing the supply of nitrate to nitrite, DNRA remained the dominant N-reduction pathway in the reactor. This is in accordance with pure culture studies by Yoon et al. (2015b). Our observations are clearly different from the observations in a chemostat enrichment culture by Kraft et al. (2014). They reported that in an electron acceptor limited marine enrichment culture, fed with glucose, acetate and amino acids, nitrate selected for a DNRA community whereas nitrite selected for a denitrifying community.

The enrichment of a DNRA culture under nitrite limitation with a nitrate based DNRA culture as inoculum was confirmed by starting a similar enrichment culture inoculated with activated sludge. In this case initially a denitrifying culture was obtained, likely due to their faster growth rate. The denitrification culture was rapidly replaced by a stable DNRA culture. This development from activated sludge inoculum replicates the enrichment of a DNRA culture under nitrate limiting conditions (van den Berg et al. 2015). This emphasizes the similarity of nitrate or nitrite in the enrichment of DNRA bacteria.

**Fig. 3:**
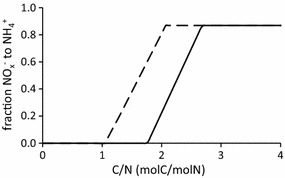
Predicted ammonium production at different influent acetate:nitrogen ratios in a chemostat fed with nitrite (*dashed line*) or nitrate (*solid line*) as electron acceptor. The ammonia production using nitrate is obtained from the model for our previous study (van den Berg et al. 2016). The shown ammonia concentrations for use of nitrite are an extrapolation of the model data

Under single substrate limiting conditions in a chemostat, Yoon et al. (2015b) observed no effect of nitrate versus nitrite on the end-product of the nitrate reduction process in their pure culture of *S. loihica* strain PV-4, using partial lactate oxidation to acetate. Like in our study, the end-product of the nitrogen conversion was predominantly ammonium under electron acceptor limiting conditions. Yoon et al. (2015b) did observe an effect of nitrite when the C/N ratio of the influent substrates was such that both electron donor and acceptor were limiting. When nitrite instead of nitrate was used under these conditions, a higher fraction of the influent nitrogen was converted to ammonium, i.e. increase of the lactate that was used for DNRA and decrease for denitrification. In our system we would expect a similar observation, due to the lower electron acceptor capacity of NO_2_^−^ compared to NO_3_^−^. In other words, more moles of electron donor are required per mole nitrate compared to nitrite. The effect of C/N ratio essentially is a result of the ratio of electrons that can be donated over electrons that can be accepted. To exemplify this, an extrapolation was done using the data and model of our previous study with the same chemostat enrichment culture system on the C/N effect (Van den Berg et al. 2016). For use of nitrite instead of nitrate, both the stoichiometry of DNRA and denitrification change. As a result the dual limitation range shifts and slightly broadens, as described in Fig. 3. For example, at a C/N ratio of 2 molC/molN with nitrate-N, the system will result in a steady state in the dual limitation phase with coexistence of both processes, whereas at this ratio of 2 molC/molN for nitrite-N, in the steady state only nitrite will be limiting and DNRA dominates. Thus, at the same C/N ratio, a change in electron acceptor from nitrate to nitrite will result in more reduction to ammonia and less denitrification to dinitrogen gas. Thus nitrite, replacing nitrate, affects the competition by changing the electron accepting capacity and thereby making the conditions more electron acceptor limited. This effect would presumably also be observed in our system, when tested under dual substrate limited conditions.

In the electron acceptor limited chemostat enrichments of Kraft et al. (2014), nitrite was predominantly reduced to dinitrogen gas, whereas the main product of nitrate reduction was ammonium. The authors observed that despite additional factors which might favor DNRA (e.g. increase of C/N ratio of the substrates, addition of sulfide, lower pH, or the use of non-fermentative electron donors), denitrification remained dominant when nitrite was supplied as electron acceptor. Therefore, Kraft et al. (2014) proposed nitrite versus nitrate as one of the key factors in the competition between denitrification and DNRA in their marine system and furthermore suggest that denitrifiers have a higher apparent affinity for nitrite and DNRA bacteria for nitrate. The results of this study and of Yoon et al. (2015b) illustrate that the effect of nitrite/nitrate supply per se is not a universal controlling factor in the competition between denitrification and DNRA. At the same time, the ambiguity shows that a combination of environmental factors can have more significant differentiating effects. As already stated by (Beijerinck 1901) we first need to establish the effect of separate environmental factors using simple systems to understand behavior in more complex lab systems.

Despite the supplementary inoculation of our established enrichment chemostat culture with fresh activated sludge from an existing wastewater treatment plant and denitrifier communities, the change of electron acceptor from nitrate to nitrite did not change the dominant ribotype in the electron acceptor limited chemostats described in this work. In all experiments the same *Geobacter lovleyi* related ribotype dominated the microbial community. Apparently, under the used conditions, this species has highest affinity (µ^max^/K_s_) for both electron acceptors.

Our results suggest that nitrite and nitrate fluctuations in an environment will have limited influence on the dominant nitrate reducing process when acetate is the electron donor. This implies that other competition affecting factors, such as pH, sulfide concentrations, or the type and complexity of the electron donor, may have a decisive effect on the nitrate reducing pathway that dominates, rather than, as suggested by Kraft et al. (2014), via direct control of either nitrite or nitrate as electron acceptor.

In summary, we show nitrite is not a controlling factor in the competition between DNRA and denitrification in a fresh water mixed culture chemostat with acetate as electron donor. In our experiments no changes were observed in the nitrogen reducing pathway when nitrate was replaced by nitrite as electron acceptor. The dominant process remained DNRA and the same *Geobacter* species was the dominant enriched organism, independent of the supply of nitrite or nitrate as electron acceptor. When starting from a fresh inoculum with nitrite as electron acceptor, DNRA outcompeted denitrification.

## Additional file

**Additional file 1.** Additional tables and figure.

## Abbreviations

Ac: acetate
DNRA: dissimilatory nitrate reduction to ammonium
VSS: volatile suspended solids

## References

[CR1] Amann RI, Binder BJ, Olson RJ, Chisholm SW, Devereux R, Stahl DA (1990). Combination of 16S rRNA-targeted oligonucleotide probes with flow cytometry for analyzing mixed microbial populations. Appl Environ Microbiol.

[CR2] Beijerinck MW (1901). Anhaufungsversuche mit Ureumbakterien. Ureumspaltung durch Urease und durch Katabolismus. Centralblatt fur Bakteriologi Parasitenkunde und Infektionskrankheiten.

[CR3] Brin LD, Giblin AE, Rich JJ (2015). Effects of experimental warming and carbon addition on nitrate reduction and respiration in coastal sediments. Biogeochemistry.

[CR4] Burgin AJ, Hamilton SK (2007). Have we overemphasized the role of denitrification in aquatic ecosystems? A review of nitrate removal pathways. Front Ecol Environ.

[CR5] Daims H, Brühl A, Amann R, Schleifer K-H, Wagner M (1999). The domain-specific probe EUB338 is insufficient for the detection of all *Bacteria*: development and evaluation of a more comprehensive probe set. Syst Appl Microbiol.

[CR6] Decleyre H, Heylen K, Van Colen C, Willems A (2015). Dissimilatory nitrogen reduction in intertidal sediments of a temperate estuary: small scale heterogeneity and novel nitrate-to-ammonium reducers. Front Microbiol.

[CR7] Giblin A, Tobias C, Song B, Weston N, Banta G, Rivera-Monroy V (2013). The Importance of dissimilatory nitrate reduction to ammonium (DNRA) in the nitrogen cycle of coastal ecosystems. Oceanography.

[CR8] Housecroft C, Sharpe A (2005) Chapter 13: the group 14 elements. In: Inorganic chemistry, 2nd edn. Pearson Prentice Hall, England, p 368

[CR9] Jetten MS (2008). The microbial nitrogen cycle. Environ Microbiol.

[CR10] Johnson K, Jiang Y, Kleerebezem R, Muyzer G, van Loosdrecht MC (2009). Enrichment of a mixed bacterial culture with a high polyhydroxyalkanoate storage capacity. Biomacromolecules.

[CR11] Kraft B, Strous M, Tegetmeyer HE (2011). Microbial nitrate respiration—genes, enzymes and environmental distribution. J Biotechnol.

[CR12] Kraft B, Tegetmeyer HE, Sharma R, Klotz MG, Ferdelman TG, Hettich RL, Geelhoed JS, Strous M (2014). The environmental controls that govern the end product of bacterial nitrate respiration. Science.

[CR13] Manz W, Amann R, Ludwig W, Wagner M, Schleifer K-H (1992). Phylogenetic oligodeoxynucleotide probes for the major subclasses of Proteobacteria: problems and solutions. Syst Appl Microbiol.

[CR14] Rütting T, Huygens D, Müller C, Van Cleemput O, Godoy R, Boeckx P (2008). Functional role of DNRA and nitrite reduction in a pristine south Chilean Nothofagus forest. Biogeochemistry.

[CR15] Rütting T, Boeckx P, Müller C, Klemedtsson L (2011). Assessment of the importance of dissimilatory nitrate reduction to ammonium for the terrestrial nitrogen cycle. Biogeosciences.

[CR16] Schäfer H, Muyzer G (2001) Denaturing gradient gel electrophoresis in marine microbial ecology. In: John HP (ed) Methods in microbiology, vol 30. Academic Press, New York, pp 425–468

[CR17] Silver WL, Herman DJ, Firestone MK (2001). Dissimilatory nitrate reduction to ammonium in upland tropical forest soils. Ecology.

[CR18] Tiedje JM, Sextone AJ, Robinson JA (1982). Denitrification: ecological niches, competition and survival. Antonie van Leeuwenhoek.

[CR19] van den Berg EM, Van Dongen U, Abbas B, Van Loosdrecht MC (2015). Enrichment of DNRA bacteria in a continuous culture. ISME J.

[CR20] van den Berg E, Boleij M, Kuenen J, Kleerebezem R, van Loosdrecht M (2016). DNRA and denitrification coexist over a broad range of acetate/N-NO_3_-ratios, in a chemostat enrichment culture. Front Microbiol.

[CR21] Vishniac W, Santer M (1957). The Thiobacilli. Bacteriol Rev.

[CR22] Yoon S, Cruz-Garcia C, Sanford R, Ritalahti KM, Loffler FE (2015). Denitrification versus respiratory ammonification: environmental controls of two competing dissimilatory NO_3_^−^/NO_2_^−^ reduction pathways in *Shewanella loihica* strain PV-4. ISME J.

[CR23] Yoon S, Sanford RA, Loffler FE (2015). Nitrite control over dissimilatory nitrate/nitrite reduction pathways in *Shewanella loihica* strain PV-4. Appl Environ Microbiol.

