# Supplementary material for: Role of nitrite in the competition between denitrification and DNRA in a chemostat enrichment culture
Source: AMB Express. 2017 May 11;7:91. doi: 10.1186/s13568-017-0398-x (PMC5425655; doi:10.1186/s13568-017-0398-x)
Supplement: Supplementary file 1 — Additional file 1. Additional tables and figure. [file 13568_2017_398_MOESM1_ESM.pdf]

AMB Express

Supplementary materials for

Role of nitrite in the competition between denitrification and DNRA in a chemostat enrichment culture

Eveline M. van den Berg, Jules L. Rombouts, J. Gijs Kuenen, Robbert Kleerebezem and Mark C. M. van Loosdrecht

Department of Biotechnology, Delft University of Technology, Delft, The Netherlands;
Van der Maasweg 9; 2629 HZ Delft, The Netherlands

Correspondence to Eveline M. van den Berg: E.M.vandenBerg@tudelft.nl

Table S1a Test with SILVA TestProbe (database SSU 128, sequence collection REFNR).

Mismatches allowed	Matches	
0	0	-
1	0	-
2	1	uncultured in genus *Geobacter*

Table S1b Test with RDP ProbeMatch.

Mismatches allowed	Matches	
0	2	uncultured in genus *Geobacter*[1]
1	4	uncultured in genus *Geobacter*
2	7	uncultured in genus *Geobacter* (6)<br>order of unclassified *Desulfuromonadales* (1)

[1] These are two sequences of the ribotype the probe specifically targets, which were deposited in our previous study (Van den Berg et al. 2015).

Table S2a. Steady state conversion rates in the reactor with average deviation.

Influent NO_2^- (N%)	Compound conversion rates (mmol h^{-1})						
	Ac^-	NO_3^-	NO_2^-	H^+	Biomass	NH_4^+	CO_2
0	-0.85 ± 0.04	-0.60 ± 0.01	-	-1.97 ± 0.05	0.46 ± 0.04	0.44 ± 0.08	1.04 ± 0.06
23	-0.80 ± 0.03	-0.45 ± 0.01	-0.13 ± 0.00	-1.77 ± 0.03	0.39 ± 0.04	0.43 ± 0.01	0.92 ± 0.06
47	-0.71 ± 0.03	-0.30 ± 0.01	-0.27 ± 0.01	-1.53 ± 0.04	0.34 ± 0.03	0.45 ± 0.01	0.88 ± 0.06
73	-0.63 ± 0.02	-0.15 ± 0.00	-0.40 ± 0.01	-1.59 ± 0.02	0.33 ± 0.03	0.43 ± 0.01	0.81 ± 0.05
100	-0.63 ± 0.04	-	-0,60 ± 0.01	-1.49 ± 0.11	0.30 ± 0.06	0.44 ± 0.02	n.a.

Table S2b. Balances over the steady state conversion rates in the reactor.

Influent NO_2^- (N%)	Balance residuals (%)		
	Carbon	Reduction	Charge
0	7	3	6
23	11	6	1
47	8	5	10
73	5	3	1
100	-	8	12

Table S3. Growth yield values in the reactor steady states, calculated from values in table A1a.

Nitrite	Biomass yield	
[N%]	[g VSS/mol acetate]	[g VSS/mol N]
0	13.4 ± 0.6	19.0 ± 0.3
23	12.2 ± 0.5	16.6 ± 0.4
47	11.8 ± 0.5	14.8 ± 0.3
73	13.0 ± 0.5	14.9 ± 0.3
100	11.7 ± 1.5	12.3 ± 1.4

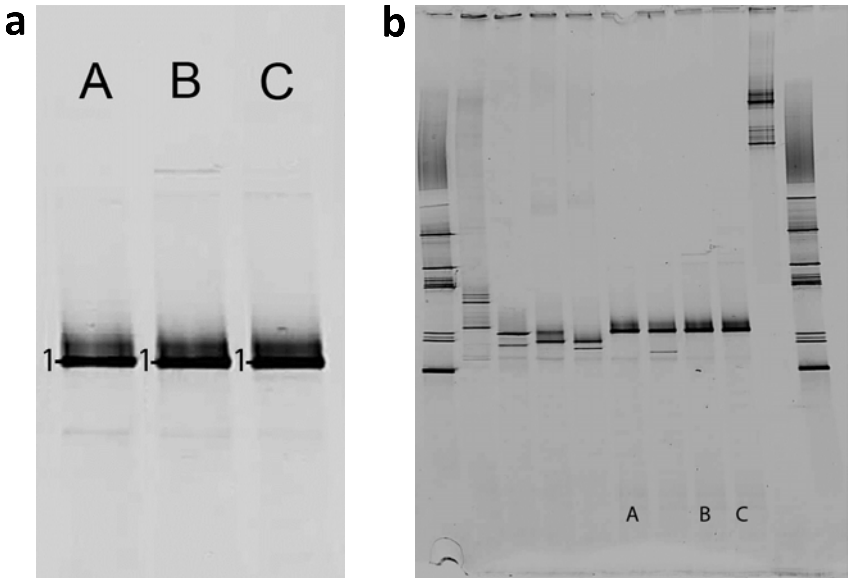

Fig. S1

(**a**) DGGE image zoomed and processed using Adobe Photoshop. (**b**) Full DGGE gel picture. The sample in lane A is from the steady state with nitrite limitation, after stepwise change from nitrate. The sample in lane B is from the steady state with limiting nitrate. The sample in lane C is from the steady state with limiting nitrite, enriched directly from activated sludge. The DGGE bands sequences of the bands indicated with 1 were identical. Additionally, they were similar to the ribotype sequences found in our previous studies (identical to NCBI accession KT317071, one gap with KM403205), which were closely related to *Geobacter lovleyi* strain SZ (Van den Berg et al. 2016; Van den Berg et al. 2015).

References

Van den Berg E, Boleij M, Kuenen J, Kleerebezem R, van Loosdrecht M (2016) DNRA and denitrification coexist over a broad range of acetate/N-NO3- ratios, in a chemostat enrichment culture Frontiers in microbiology 7 doi:10.3389/fmicb.2016.01842

Van den Berg EM, Van Dongen U, Abbas B, Van Loosdrecht MC (2015) Enrichment of DNRA bacteria in a continuous culture The ISME journal 9:2153-2161 doi:10.1038/ismej.2015.26
